# Advances in Understanding and Managing Myasthenia Gravis: Current Trends and Future Directions

**DOI:** 10.7759/cureus.59104

**Published:** 2024-04-26

**Authors:** Shreya Jaiswal, Tripti Shrivastava

**Affiliations:** 1 Medicine, Jawaharlal Nehru Medical College, Datta Meghe Institute of Higher Education and Research, Wardha, IND; 2 Physiology, Jawaharlal Nehru Medical College, Datta Meghe Institute of Higher Education and Research, Wardha, IND

**Keywords:** thymoma, muscle, thoracoscopic thymectomy, myasthenia gravis, autoimmune illness, neuromuscular junction, thymectomy

## Abstract

Myasthenia gravis (MG) is an autoimmune illness characterized by autoantibodies against the acetylcholine receptor (AChR), muscle-specific tyrosine kinase (MuSK), and an increasing number of extra postsynaptic proteins. Pathogenic autoantibodies reduce the number of functional AChRs in the neuromuscular junction's (NMJ) muscle end plate. The cause of the autoimmune response is unknown, but thymic abnormalities and immune regulatory deficiencies are significant. The disease's incidence is likely influenced by genetic predisposition, with sex hormones and exercise playing a role. MG can affect any age, race, or gender and can be caused by any stressor, with infections being the most frequent cause. Treatment focuses on airway support and the triggering incident. MG is a rare autoimmune disease causing fatigue-inducing weakness in the axial, respiratory, leg, and bulbar muscles. Initially affecting the eyes, most MG patients experience at least one worsening symptom during their illness. The disease is mainly caused by antibodies against the AChR, dependence on the immune system within cells, and engagement of the complement system. The complement system plays a significant role in MG, and complement inhibition can both prevent the onset and slow its development. Ocular MG affects around 15% of people, with most patients having blocking antibodies against the cholinergic receptor. There may be correlations between thymoma and other autoimmune conditions, especially thyroid illness. Treatment and management for MG involve removing autoantibodies from circulation or blocking effector mechanisms using techniques such as complement inhibition, plasmapheresis, and B-cell elimination.

## Introduction and background

Myasthenia gravis (MG) is a rare autoimmune disorder characterized by autoantibodies against certain proteins. Globally, its prevalence ranges from 20 to 200 cases per million people, with an annual incidence of one to nine cases per million. MG predominantly affects women under 40 and men over 60, though it can occur at any age. Factors like age, sex, genetics, and geography influence its prevalence and incidence. The disease acts through B-cells [1]. The thymus is a critical player in etiopathogenesis, and the acetylcholine receptor (AChR) is the target of most pathogenic autoantibodies found. Over the past 20 years, there has been a significant advancement in our understanding of the immunological components of the neuromuscular junction (NMJ). This has made it possible to identify new pathogenic antibodies, lower the number of patients suffering from seronegative myasthenia, and suggest patient subgroups with potential clinical and therapeutic benefits [2]. Most people with MG initially exhibit symptoms related to their eyes. The majority of MG patients will go through at least one episode of symptom aggravation during their disease. This article will discuss the natural history, categorization, clinical presentation, and epidemiology of MG [3].

The clinical features, cholinesterase inhibitor efficacy, and finding of specific autoantibodies (such as anti-AChR, anti-MuSK, or anti-lipoprotein receptor-related protein 4 (Lrp4)), as well as a notable decline demonstrated by electrophysiological testing, all play a role in the diagnosis. We provide a brief overview of the disease's history, epidemiology, diagnostic, and clinical categorization in this study [4]. New therapies are being explored; the outcomes of the thymectomy randomized controlled trial in MG, scheduled for publication in early 2016, will be precious from a therapeutic standpoint. In the areas of pregnancy, ocular, and generalized myasthenia gravis (GMG), practicing physicians may refer to recent MG guidelines for guidance in navigating an evidence base of differing quality [5]. None of these individuals had a thymoma that was apparent. These antibodies, primarily of the IgG1 subclass, prevent Lrp4 from binding to its ligand. In other studies using Lrp4 ab, sera positive for Lrp4 ab, prevented AChRs from aggregating in cultured myotubes when exposed to agrin, indicating a pathogenic involvement in the malfunction of the neuromuscular endplate [6]. NMJ disorders include autoimmune, toxic, and hereditary illnesses. They are characterized by varying and fatigue-able weakness. The majority of adult NMJ illnesses are antibody-mediated; MG is the most prevalent, affecting around one in 10,000 people, with a roughly 2:1 gender disparity in cases. The advancements in the treatment of autoimmune MG and LEMS are the main topic of this essay [7]. This overview covers the natural history of MG, clinical signs, diagnosis, and available treatments. The myasthenic syndrome, also known as Lambert-Eaton syndrome, is a rarer autoimmune condition caused by antibodies that target PQ-type voltage-gated calcium channels.

Treatment alternatives and clinical characteristics are enumerated [8]. MG is not commonly seen in the practice of pediatric ophthalmology. McCreery's KMB study aims to assess the clinical spectrum of this disorder in children and to pinpoint potential helpful variables for the diagnosis and treatment of the condition [9]. Fifteen international experts in MG participated in the consensus process. In the context of a medical consensus process, experts typically come together to establish agreements or guidelines on various aspects of a disease. This can include aspects such as diagnostic criteria, treatment protocols, management strategies, prognostic factors, and research priorities. For instance, in the case of MG, a consensus process might focus on agreeing upon standardized methods for diagnosis, defining optimal treatment approaches, identifying indicators of disease severity, or determining criteria for assessing treatment response [10]. Treatment plans are frequently tailored based on a patient's medical history, comorbidities, antibody status, and other variables. This review provides current, high-yield, clinically relevant information on MG using a question-and-answer style [11]. Variable and fatigable weakness affecting the ocular muscles (producing diplopia and ptosis), the bulbar muscles (causing dysphagia, dysarthria, and dyspnea), and the extremities muscles are the disease's clinical signature. Serological testing that detects either muscle-specific tyrosine kinase (MuSK) antibodies or AChR antibodies is most frequently used to make the diagnosis. The diagnosis is supported in part by electrodiagnostic testing. Numerous therapies are available that lead to improvements in life quality and function [12]. In MG treatment, tacrolimus is a second-line immunosuppressant that is mostly used in conjunction with corticosteroids to lower steroid dosage and preserve the immunotherapy's effects. However, there are not many trials that specifically examine tacrolimus' role in attaining minimum symptom status as a single immunotherapy drug [13]. Due to the erratic kind of muscle weakness and how its symptoms and indicators coincide with those of other neuromuscular illnesses, diagnosing MG can be difficult and takes some time. This page discusses the significance of quickly recognizing the usual symptoms and indications, the best tests to confirm the diagnosis, the function of thymectomy, the available acute and chronic treatment options, and the disease's natural history. We will also go over special considerations for the diagnosis and treatment of pregnant women and children. There is also a summary of congenital myasthenic syndromes in this article [14]. Due to the erratic nature of muscle weakness and the overlap of signs and symptoms with other neuromuscular illnesses, diagnosing MG can be difficult and time-consuming. This article covers the natural history of the disease, the goal of a thymectomy, the best tests to confirm the diagnosis, the significance of quickly recognizing the usual symptoms and signs, the available acute and chronic treatment options, and the procedure. We will also discuss unique concerns for diagnosing and treating youngsters and pregnant women. This page also includes a synopsis of congenital myasthenic syndromes [15].

## Review

Search methodology

**Figure 1:**
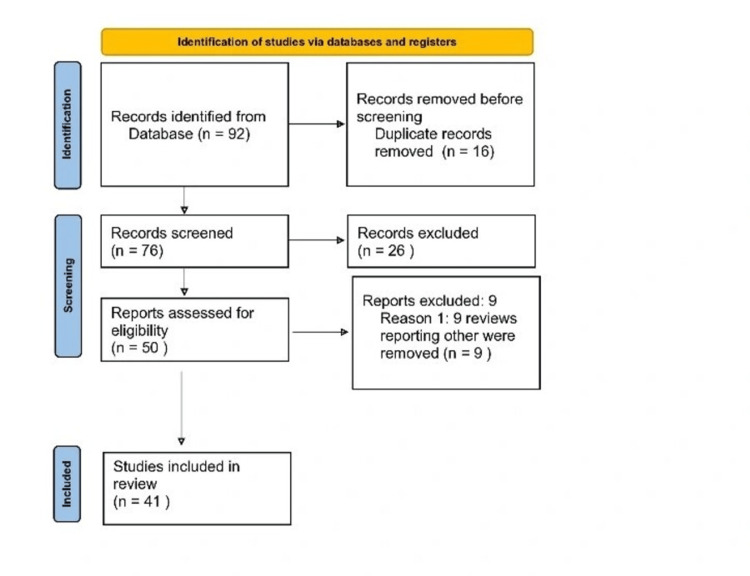
PRISMA flow chart PRISMA, preferred reporting items for systematic reviews and meta-analyses

We systematically searched PubMed from April to May 2023, employing keywords such as NMJ, MG, autoimmune disease, thymectomy, muscle, and thymoma. Initially, one reviewer independently assessed the first retrieved paper and its abstract against the inclusion criteria before reviewing the full text. Finally, 41 articles were included in the review. Figure 1 shows the literature search strategy.

Etiology and pathophysiology

MG stands as a prime illustration of an autoimmune disorder. Autoantibodies targeting the NMJ characterize this condition. Initially, the majority of individuals with MG manifest symptoms predominantly related to ocular involvement. Throughout the course of the disease, most MG patients experience at least one episode of symptom exacerbation. This article aims to delve into the natural progression, classification, clinical manifestations, and epidemiology, which collectively characterize this disorder. Pathogenic autoantibodies result in a reduction in the number of functional AChRs or a functional interruption of the AChR in the muscle end plate of the NMJ. There is still much to learn about the cellular immunology that contributes to generating autoantibodies, even though the molecular immunology underlying the pathophysiology is well established [16]. One renowned instance of an autoimmune disorder is MG, where the majority of individuals initially present symptoms associated with ocular involvement. Most MG patients experience at least one episode of symptom exacerbation during the course of their illness. This article aims to explore the natural progression, classification, clinical presentation, and epidemiology of MG. The decline or functional disruption of functional AChRs at the NMJ muscle end plate occurs due to the presence of detrimental autoantibodies. There is still much to learn about cellular immunology, even though the molecular immunology underlying the pathophysiology is well recognized, which plays a role in the production of autoantibodies [17].

Although the cause of the autoimmune response is unknown, thymic abnormalities and immune regulatory deficiencies undoubtedly play a significant role in anti-acetylcholine receptor antibodies (AChR-Ab)-positive individuals. The disease's incidence is likely influenced by genetic predisposition. The early stage of the illness appears to be influenced by sex hormones. Exercise makes muscle weakness worse and more erratic. MG patients can be categorized based on the autoantibody profile, thymic abnormalities, age at onset of symptoms, and location of affected muscles (ocular versus generalized) [18]. Any age, race, or gender can be affected by MG, which can be brought on by any stressor, with infections being the most frequent cause. The clinical diagnosis of MG is characterized by respiratory failure brought on by an MG flare-up. Any voluntary muscle might become weak. Normal reflexes, sensibility, autonomic symptoms, fasciculations, and progressive weakness with repeated motion distinguish MG from other NMJ illnesses. The goal of treatment should be airway support and the triggering incident [19]. Antibodies against the lipoprotein receptor protein 4, the MuSK, and the AChR identify illness subtypes with different immunological-pathogenic processes and clinical characteristics. Genes in the immune response have susceptibility loci found within them thanks to genome-wide methods [20].

Clinical presentation and diagnosis

Despite being an uncommon condition, MG is the most prevalent NMJ dysfunction. It is estimated to affect between 14 to 20 individuals per 100,000 people. However, these rates can differ significantly based on factors such as age, gender, ethnicity, and geographic location. It is the quintessential autoimmune illness, usually brought on by antibodies to the AChR. It leads to distinct, fatigable weakness in the leg, respiratory, bulbar, ophthalmic, and axial muscles. The majority of individuals with MG initially present symptoms primarily associated with their eyes. Throughout the course of the disease, most MG patients experience at least one episode of symptom exacerbation [21]. MG is a rare disorder, It is a principal autoimmune disorder typically triggered by antibodies targeting the AChR, leading to characteristic fatigue-inducing weakness in the bulbar, respiratory, leg, and axial muscles. Initially, the majority of individuals with MG present symptoms linked to ocular involvement. Throughout the course of the illness, most MG patients will encounter at least one episode of symptom exacerbation. MG, the autoimmune condition, primarily arises due to the presence of AChR-Ab, involving cellular immunological mechanisms and the complement system [22].

An immune onslaught destroys the AChR on the postsynaptic membrane, making it unable to generate enough endplate potential. This leads to a synaptic transmission problem at the NMJ and muscle weakness. Clinical trials and animal models have shown the significance of the complement system in MG, and it has been found that complement inhibition in MG patients can both prevent the onset of the illness and slow its development [23].

Ocular MG affects around 15% of people; symptoms are limited to this distribution. Most patients have blocking antibodies against the cholinergic receptor; however, a lesser percentage of patients have antibodies against other similar targets, which are linked to specific phenotypes. There may be correlations between thymoma and other autoimmune conditions, especially thyroid illness [24]. These articles include a tabulation of the authors, journal, country and date of publication, study type, patient group under investigation, and relevant outcomes and findings. Complete stable remission (CSR), pharmacological remission, age at presentation, gender, symptom duration, preoperative classification (Oosterhius, Osserman, or Myasthenia Gravis Foundation of America (MGFA)), thymic pathology, preoperative medications (steroids, immunosuppressants), mortality, and morbidity were among the outcome variables that were consistent across all of the papers. We find that evaluations based on data have demonstrated that patients who have had a thymectomy have a higher chance of becoming asymptomatic, achieving medication-free remission, and improving clinically (54%, P<0.01) than patients who have not had a thymectomy, particularly if they have severe and widespread symptoms (P=0.007). Patients differ significantly in their clinical appearance and pathology; some have a weakness that is restricted to the ocular muscles, while others have a substantial generalized weakness that results in respiratory insufficiency. MG is an antibody-mediated illness that depends on B-cells that are autoreactive and need T-cell assistance. Treatments concentrate on removing autoantibodies from circulation or blocking effector mechanisms using various techniques, such as complement inhibition, plasmapheresis, and B-cell elimination [25].

Treatment and management

A cell-directed, antibody-mediated autoimmune assault on the nicotinic AChR or, occasionally, other postsynaptic antigens is the etiology of MG. The antibodies destroy the postsynaptic endplate membrane through complement-mediated means, which lowers endplate AChR. They also partially work by increasing endplate AChR turnover or inhibiting AChR function [26]. Its usage in MG is motivated by venous access, easily accessible IgG (until recently), and the comparatively low number of significant, observable side effects. Its efficacy in adults has been patchy due to erratic AChR antibody responses. Ten kids had their clinical reactions to high-dose intravenous immunoglobulin (HDIVIG) and its side effects assessed. Three patients had weekly anti-AChR antibody titers measured. In one patient, the total dose was 0.8 gm/kg to adjust to the appropriate body weight. The HDIVIG dosage was 2 gm/kg body weight, infused at varying rates of 2 gm/kg for one day, 0.66 gm/kg daily for three days, and 0.5 gm/kg daily for four days. All but one of the kids handled HDIVIG without any issues. Following HDIVIG, eight patients showed a discernible increase in their functional strength but a decline. For anesthetists, the management implications of this condition in pregnant women are pretty tricky [27].

The goal is to draw attention to some difficulties encountered, the strategies employed, and the lessons discovered while caring for this patient. This is a case study of a 31-year-old parturient who had a diagnosis of hepatitis B and MG and who was scheduled for a cesarean section. Bupivacaine was used as a single-shot spinal anesthetic throughout the procedure. Neostigmine infusion was used to control intraoperative myasthenia crises. After receiving care for a few days in the intensive care unit, she was released. She had severe dyspnea and widespread musculoskeletal weakness while under spinal anesthesia, but her hemodynamics remained stable throughout the procedure [28]. An autoimmune condition called MG mainly affects women who are of reproductive age. Though more challenging to treat early in gestation, the disease's progress during pregnancy is uncertain. Antibodies to the muscle-specific receptor tyrosine kinase (anti-MuSK) have been reported as a subgroup of MG with more localized clinical symptoms and a worse response to therapy than individuals who have antibodies to the anti-AChR [29]. His clinical and electrophysiologic test findings, the following temporal profile of the ptosis, and a history of a similar episode that occurred briefly two years prior led to the diagnosis of very moderate, generalized, antibody-negative MG. His want to play football further created several unresolved management issues for which there was no documented instruction. We began daily high-dose prednisone medication with potassium and calcium supplements, and we let him engage in conditioning activities without physical contact. A varied illness, MG, presents with a changing clinical, pathological, and immunobiological picture [30].

These days, it is thought that immunosuppression and surgery are necessary for MG treatment to be effective. Neurologists propose thymectomy as a treatment option for individuals with nontumoral MG to improve or achieve remission. Nowadays, thoracoscopic thymectomy is regarded as a good substitute for the open standard method because of its remarkable effectiveness, as indicated by total stable remission rates, minimal morbidity, and better acceptance rate. We provide an overview of the experience with unilateral prolonged thoracoscopic thymectomy for nontumoral MG. This procedure can potentially establish a new benchmark in the intricate therapeutic management of MG [31]. The hereditary illnesses known as congenital myasthenia syndromes are diverse and are typified by impaired neuromuscular transmission. Despite sharing muscular fatigability with acquired immune myasthenia syndrome, there are significant distinctions in their pathophysiology, diagnosis, treatment, and development patterns. We describe the care of a 28-year-old woman who had an elective cesarean birth performed under spinal anesthesia and had long-standing congenital myasthenia syndromes. Her care was exacerbated by muscle imbalance, scoliosis caused by weakness, and recurrent respiratory failure. The spinal anesthetic was made more accessible with the assistance of ultrasonography. Effective lung volume maintenance and prevention of respiratory function decline were achieved with intraoperative noninvasive positive pressure ventilation [32]. An immune system that is dysregulated and unable to distinguish between self and non-self antigens is a prevalent pathogen in autoimmune disorders. The intricate interactions between hereditary and environmental variables significantly influence the disease's onset and progression. Disrupted gut microbiota, or gut dysbiosis, is one of the environmental variables that has received a lot of attention lately, especially with the advancements in human microbiome research. There is a shortage of knowledge regarding disorders of the neuromuscular system, even though changes in microbiota have been seen in several autoimmune illnesses, including nervous system disorders. One such uncommon autoimmune NMJ illness is MG, which is brought on by producing harmful autoantibodies against elements of the postsynaptic muscle endplate [33].

Today's clinical practice shows a move towards ICU-acquired weakness despite primary neuromuscular illnesses being the most prevalent source of weakness from peripheral nervous system disease in the I.C.U. Determining the source of weakness is crucial since it might have a significant impact on prognosis. The most prevalent primary neuromuscular illnesses in the intensive care unit are still Guillain-Barré syndrome and MG. It is critical to monitor the airway closely and start noninvasive ventilation early in the course of the illness. Conversely, in order to prevent more problems, individuals with Guillain-Barré syndrome should have their airway intubated as soon as possible [34]. An autoimmune condition called MG affects the NMJ and is typified by aberrant fatigability and varying degrees of muscle weakening. For individuals with MG, the use of muscle relaxants during anesthesia is a significant problem. A valuable tool for ensuring immobilization during general anesthesia surgery is a muscle relaxant. Without muscle relaxants, anesthetic care for MG patients is a challenge since immobilization is not always guaranteed. However, in patients with MG, the pharmacological effects of muscle relaxants might last longer, which raises the need for postoperative respiratory assistance. Here, we detail the anesthetic care of a man with MG, age 82, who is having laparoscopic surgery [35].

Thymectomy

In adult MG, thymectomy is a well-established therapy; however, its precise function in JMG is still unknown. When treating severe JMG that is not responding to treatment, thymectomy is often recommended. 

Indeed, certain patient characteristics may predict a more favorable response to thymectomy in the treatment of severe JMG that is not responding to other therapies. Some factors associated with a better outcome post-thymectomy include age, younger age at the time of thymectomy is often linked with better treatment response; disease duration, patients with shorter disease duration tend to have more favorable outcomes; presence of thymoma, thymectomy is particularly effective in patients with thymoma-associated JMG; antibody status, some studies suggest that patients with AChR-Ab-positive status may have better responses to thymectomy; and histological findings, thymectomy is more beneficial in patients with thymic hyperplasia compared to those with thymic atrophy.

From open median sternotomy to the more cosmesis-preserving thoracoscopic technique, surgical techniques have changed. This research examines the available data about the efficacy of thymectomy in JMG patients and discusses clinical traits that could be linked to better results. There were 17 research with 588 individuals who had thymectomy between 1997 and 2020; these studies either reported on uncontrolled cohorts having thymectomy or compared cohorts having various surgical techniques [36]. One of the treatment options for MG is thymectomy. Over the last eight decades, several observational studies have demonstrated that thymectomy may expedite the disease's stabilization, lessen the corticosteroid requirement, and, in some instances, result in total remission. Propensity score analysis supports the effectiveness of thymectomy in MG. The results of a recently concluded randomized control study investigating the function of thymectomy in individuals with non-thymomatous MG have not yet been published. There is still much debate over the method and scope of thymectomy, especially in light of the advancements in minimally invasive surgery [37].

Prognosis and quality of life

Context thymoma is a primary tumor that often appears in the mediastinum and is generated from the thymus's epithelial cells. The most drastic method of treating thymoma is surgical thymectomy. The quality of thymectomy surgery has increased with the advent of video-assisted thoracoscopic surgery. There is currently a shortage of information about the clinical characteristics of thymoma and the outcome of thoracoscopic thymectomy in Vietnamese patients using video assistance. The objectives of this study were to examine the clinical and biochemical features of thymoma and evaluate the initial results of video-assisted thoracoscopic thymectomy for thymoma in Vietnamese patients. The study comprised all 53 patients with thymoma, regardless of whether they had MG, who had video-assisted thoracoscopic thymectomy at Military Hospital 103 in Vietnam between October 2013 and July 2017 [38]. Using information from two recently concluded treatment studies for GMG, the results of the MMT (Manual Muscle Test), MG-ADL (myasthenia gravis-activities of daily living), and quantitative myasthenia gravis (QMG) scales. Items pertinent to MG symptoms, significant to the patient and the doctor, and adaptable to clinical change were chosen. Following the selection of the 10 measures, the items were weighted according to feedback from global MG specialists, taking into account aspects including validity, reliability, risk, prognosis, and quality of life in addition to illness severity and risk. The MG composite requires no special equipment, is simple to administer, and may be finished in less than five minutes [39].

This study was conducted because there is a shortage of research on the causes of extended breathing, myasthenic crisis (MC), and their predictors. There were 64 MG patients, with a median age of 45 (six to 84) years. They were seen to have a thymectomy, AChR-Ab, thymoma, comorbidities, offending medications, and MC. It was noted who needed ventilation for more than 15 days. We collected data on hospital mortality, MG quality of life (QOL) at discharge, and yearly hospital visits, admissions, expenses, and missed work days. After a median follow-up of 48 (three to 264) months, 14 (21.9%) patients had MC within one to 120 (median: 8.5) months after the illness began [40]. The most frequent tumors of the mediastinum are thymomas. Patients with thymomas suffer significantly from these tumors, which frequently compress vital mediastinal organs. Some of our patients with incurable malignant thymomas have chosen to have cryotherapy to escape the adverse effects of chemoradiotherapy. Examining our hospital's cryosurgery, nursing, and follow-up data during the previous eight years, we assessed the efficacy and safety of cryotherapy for 19 patients with incurable malignant thymomas. No severe side effects from cryosurgery affected the surrounding vital organs of the tumor [41]. Table 1 details the findings of the articles included in the review. 

**Table 1:** Characteristics of articles included in the review AChR, acetylcholine receptor; MG, myasthenia gravis; GMG, generalized myasthenia gravis; NMJ, neuromuscular junction; AChR-Ab, acetylcholine receptor antibodies; MGFA, Myasthenia Gravis Foundation of America

Authors	Year	Findings
Gilhus NE et al. [1]	2015	There are more immunomodulatory medications on the horizon, but the lack of controlled trials makes treatment choices difficult. For the majority of patients, long-term medication therapy is necessary and has to be customized for their specific kind of MG.
Pardo Fernandez et al. [2]	2023	MG is an autoimmune illness characterized by fatigueable muscle weakening. It is produced by unique antibodies that target distinct postsynaptic components of the NMJ.
Berrih Aknin S et al. [4]	2014	Ptosis and diplopia are the typical initial signs of the disease, and in 80% of instances, it spreads to other muscles.
Tzartos J et al. [5]	2014	However, in this instance, they are primarily IgG4 subclass, indicating that a distinct pathogenic mechanism than AChR-MG is in operation.
Ohta R et al. [6]	2014	More data is included in the MG preliminary version than in any one MG-specific tool, especially regarding the environmental aspects component.
Pascuzzi RM [8]	2002	Pathogenic antibodies against the AChR are a typical feature of the autoimmune disease MG.
McCreery KMB et al. [9]	2002	They reflected on the anesthetic experience with MG at Vancouver General Hospital during the previous ten years.
Gwathmey KG et al. [12]	2015	Numerous therapies are available that lead to improvements in life quality and function.
Duan W et al. [13]	2021	The postintervention state was used by the MGFA to assess the therapy impact. Using Cox regression analysis, clinical parameters influencing accomplishment and treatment reactivity of several MG subtypes were identified.
Howard JF et al. [15]	2017	Refractory GMG is probably influenced by complement, yet there are currently no licensed treatments that target this system explicitly.
Guptill JT et al. [16]	2018	There is still much to learn about the cellular immunology that contributes to generating autoantibodies, even though the molecular immunology underlying the pathophysiology is well established.
Gilhus NE et al. [17]	2016	Therapy might include symptomatic medication therapy, immunosuppressive medication therapy, thymectomy, and supportive therapy; it should be customized for each patient and directed by the MG subgroup.
Le Panse R et al. [18]	2014	Exercise makes muscle weakness worse and more erratic. The location of the afflicted muscles (ocular versus generalized), the age at which symptoms first appeared, thymic abnormalities, and the autoantibody profile can all be used to categorize cases with MG.
Roper J et al. [19]	2017	Any age, race, or gender can be affected by MG, which can be brought on by any stressor, with infections being the most frequent cause. The clinical diagnosis of MG is characterized by respiratory failure brought on by an MG flare-up. Any voluntary muscle can be weak.
Evoli A [20]	2017	An uncommon neuromuscular transmission ailment called MG is becoming more often recognized as a syndrome than as a single illness.
Xiao H et al. [23]	2021	Clinical trials and animal models have shown the significance of the complement system in MG, and it has been found that complement inhibition in MG patients can both prevent the onset of the illness and slow its development.
Shuey NH [24]	2022	This results in skeletal muscular weakness that can be erratic and fatigue-prone. It frequently presents as ptosis and diplopia, with ocular symptoms in 60% of cases initially. As a result, patients may first see eye care professionals.
Michon AM et al. [27]	2000	Out of the three patients studied, there was a consistent drop in anti-AChR antibody levels only in one patient. There was no discernible relationship between antibody titers and clinical response.
Tomulescu V et al. [31]	2012	Neurologists propose thymectomy as a treatment option for individuals with nontumoral MG to improve the likelihood of remission.
Hartley L et al. [36]	2021	Better surgical outcomes may be associated with early intervention, intervention after the onset of puberty, being AChR-Ab positive, having more severe disease, and hyperplastic thymic tissue.

## Conclusions

The amount of functional AChR in the NMJ's muscle end plate decreases or is functionally interrupted due to pathogenic autoantibodies. The early stage of the illness appears to be influenced by sex hormones. MG is the most frequent NMJ dysfunction but is a primary autoimmune illness that is typically brought on by antibodies against the AChR, which results in distinctive, fatigue-inducing weakening in the axial respiratory, leg, and bulbar muscles. A cell-directed, antibody-mediated autoimmune assault on the nicotinic AChR or, occasionally, other postsynaptic antigens is the etiology of MG. The antibodies destroy the postsynaptic endplate membrane through complement-mediated means, which lowers endplate AChR. One of the treatment options for MG is thymectomy. The results of a recently concluded randomized control study investigating the function of thymectomy in individuals with non-thymomatous MG have not yet been published. The most drastic method of treating thymoma is surgical thymectomy. The quality of thymectomy surgery has increased with the advent of video-assisted thoracoscopic surgery. 

## References

[1] Gilhus NE, Verschuuren JJ (2015). Myasthenia gravis: subgroup classification and therapeutic strategies. Lancet Neurol.

[2] García Estévez DA, Pardo Fernández J (2023). Myasthenia gravis. Update on diagnosis and therapy. Med Clin (Barc).

[3] Hehir MK, Silvestri NJ (2018). Generalized Myasthenia Gravis: classification, clinical presentation, natural history, and epidemiology. Neurol Clin.

[4] Berrih-Aknin S, Frenkian-Cuvelier M, Eymard B (2014). Diagnostic and clinical classification of autoimmune myasthenia gravis. J Autoimmun.

[5] Zisimopoulou P, Evangelakou P, Tzartos J (2014). A comprehensive analysis of the epidemiology and clinical characteristics of anti-LRP4 in myasthenia gravis. J Autoimmun.

[6] Raggi A, Schiavolin S, Leonardi M, Antozzi C, Baggi F, Maggi L, Mantegazza R (2014). Development of the MG-DIS: an ICF-based disability assessment instrument for myasthenia gravis. Disabil Rehabil.

[7] Bodkin C, Pascuzzi RM (2021). Update in the management of myasthenia gravis and Lambert-Eaton myasthenic syndrome. Neurol Clin.

[8] Pascuzzi RM (2002). Myasthenia gravis and Lambert-Eaton syndrome. Ther Apher.

[9] Grant RP, Jenkins LC (1982). Prediction of the need for postoperative mechanical ventilation in myasthenia gravis: thymectomy compared to other surgical procedures. Can Anaesth Soc J.

[10] Sanders DB, Wolfe GI, Narayanaswami P (2018). Developing treatment guidelines for myasthenia gravis. Ann N Y Acad Sci.

[11] Morren JA, Li Y (2023). Myasthenia gravis: frequently asked questions. Cleve Clin J Med.

[12] Gwathmey KG, Burns TM (2015). Myasthenia gravis. Semin Neurol.

[13] Duan W, Peng Y, Jin W, Ouyang S, Yang H (2021). Tacrolimus as single-agent immunotherapy and minimal manifestation status in nonthymoma myasthenia gravis. J Immunol Res.

[14] Ciafaloni E (2019). Myasthenia gravis and congenital myasthenic syndromes. Continuum (Minneap Minn).

[15] Howard JF, Utsugisawa K, Benatar M (2017). Safety and efficacy of eculizumab in anti-acetylcholine receptor antibody-positive refractory generalised myasthenia gravis (REGAIN): A phase 3, randomised, double-blind, placebo-controlled, multicentre study. Lancet Neurol.

[16] Yi JS, Guptill JT, Stathopoulos P, Nowak RJ, O'Connor KC (2018). B cells in the pathophysiology of myasthenia gravis. Muscle Nerve.

[17] Gilhus NE, Skeie GO, Romi F, Lazaridis K, Zisimopoulou P, Tzartos S (2016). Myasthenia gravis - autoantibody characteristics and their implications for therapy. Nat Rev Neurol.

[18] Berrih-Aknin S, Le Panse R (2014). [Myasthenia gravis and autoantibodies: pathophysiology of the different subtypes]. Rev Med Interne.

[19] Roper J, Fleming ME, Long B, Koyfman A (2017). Myasthenia gravis and crisis: evaluation and management in the emergency department. J Emerg Med.

[20] Evoli A (2017). Myasthenia gravis: new developments in research and treatment. Curr Opin Neurol.

[21] Hehir MK 2nd, Li Y (2022). Diagnosis and management of myasthenia gravis. Continuum (Minneap Minn).

[22] Martínez Torre S, Gómez Molinero I, Martínez Girón R (2018). [An update on myasthenia gravis]. Semergen.

[23] Xiao H, Wu K, Liang X, Li R, Lai KP (2021). Clinical efficacy and safety of eculizumab for treating myasthenia gravis. Front Immunol.

[24] Shuey NH (2022). Ocular myasthenia gravis: a review and practical guide for clinicians. Clin Exp Optom.

[25] Wang S, Breskovska I, Gandhy S, Punga AR, Guptill JT, Kaminski HJ (2018). Advances in autoimmune myasthenia gravis management. Expert Rev Neurother.

[26] Richman DP, Agius MA (2003). Treatment principles in the management of autoimmune myasthenia gravis. Ann N Y Acad Sci.

[27] Selcen D, Dabrowski ER, Michon AM, Nigro MA (2000). High-dose intravenous immunoglobulin therapy in juvenile myasthenia gravis. Pediatr Neurol.

[28] Hanson JA, Lueck CJ, Thomas DJ (1996). Myasthenia gravis presenting with stridor. Thorax.

[29] Neves AR, Monteiro P, Matos A, Santos Silva I (2015). Anti-MuSK-positive myasthenia gravis diagnosed during pregnancy: new challenges for an old disease?. BMJ Case Rep.

[30] Leddy JJ, Chutkow JG (2000). Myasthenia gravis in a collegiate football player. Med Sci Sports Exerc.

[31] Tomulescu V, Popescu I (2012). Unilateral extended thoracoscopic thymectomy for nontumoral myasthenia gravis—a new standard. Semin Thorac Cardiovasc Surg.

[32] Terblanche N, Maxwell C, Keunen J, Carvalho JC (2008). Obstetric and anesthetic management of severe congenital myasthenia syndrome. Anesth Analg.

[33] Kapoor B, Gulati M, Gupta R, Singla RK (2023). Microbiota dysbiosis and myasthenia gravis: do all roads lead to Rome?. Autoimmun Rev.

[34] Greene-Chandos D, Torbey M (2018). Critical care of neuromuscular disorders. Continuum (Minneap Minn).

[35] Koda K, Kimura H, Uzawa M, Sambe N, Sugano T, Kitamura T, Tagami M (2008). [Desflurane anesthesia without muscle relaxant for a patient with myasthenia gravis undergoing laparoscopic high anterior resection: a case report]. Neurotherapeutics.

[36] Ng WC, Hartley L (2021). Effectiveness of thymectomy in juvenile myasthenia gravis and clinical characteristics associated with better outcomes. Neuromuscul Disord.

[37] de Perrot M, McRae K (2017). Evidence for thymectomy in myasthenia gravis: getting stronger?. J Thorac Cardiovasc Surg.

[38] Nguyen GT, Nguyen TN, Nguyen NV, Nguyen KT, Le AV (2018). Video-assisted thoracoscopic thymectomy for thymoma: a single-center experience. Asian Cardiovasc Thorac Ann.

[39] Burns TM, Conaway MR, Cutter GR, Sanders DB (2008). Construction of an efficient evaluative instrument for myasthenia gravis: the MG composite. Muscle Nerve.

[40] Kalita J, Kohat AK, Misra UK (2014). Predictors of outcome of myasthenic crisis. Neurol Sci.

[41] Zhang Z, Wu B, Niu L (2013). Combination percutaneous cryotherapy and iodine-125 seed implantation for unresectable malignant thymoma: experience in 19 patients. Cryobiology.

